# Construct validation and correlates of preoperative expectations of postsurgical recovery in persons undergoing knee replacement: baseline findings from a randomized clinical trial

**DOI:** 10.1186/s12955-017-0810-x

**Published:** 2017-12-01

**Authors:** Daniel L. Riddle, James Slover, Dennis Ang, Robert A. Perera, Levent Dumenci

**Affiliations:** 1 Departments of Physical Therapy, Orthopaedic Surgery and Rheumatology, Virginia Commonwealth University, Richmond, Virginia 23298 USA; 2 Associate Professor, Adult Reconstructive Division, Department of Orthopaedic Surgery, NYU Hospital for Joint Diseases, 301 East 17th Street; Suite 213, New York, NY 10003 USA; 3 Department of Medicine, Section of Rheumatology, Wake Forest School of Medicine, Winston-Salem, North Carolina 27157 USA; 4 Department of Biostatistics, Virginia Commonwealth University, Richmond, VA 23298 USA; 5 Department of Epidemiology and Biostatistics, Temple University, Philadelphia, PA 19122 USA

**Keywords:** Knee, Arthroplasty, Expectations, Outcome

## Abstract

**Background:**

A patient’s recovery expectations prior to knee arthroplasty influence postsurgical outcome and satisfaction but a unidimensional measure of expectation has not been reported in the literature. Our primary purpose was to determine the extent to which a patient expectations scale reflects a unidimensional construct. Our second purpose was to identify pre-operative variables associated with patients’ expectations. We hypothesized that previously identified predictors of the latent expectation scale score would be associated with expectations and that previously unexplored variables of pain catastrophizing, depressive and anxiety symptoms, self-efficacy and number of painful body regions would also associate with pre-operative expectations.

**Methods:**

Our randomized clinical trial had 384 patients assessed prior to knee replacement surgery. The expectations scale along with several predictor variables including WOMAC, psychological distress, and sociodemographic variables were obtained. Confirmatory factor analysis tested the unidimensionality of the measure and structural equation modeling identified predictors of the latent expectations measure.

**Results:**

The expectations scale was found to be unidimensional with superior model fit (χ2 = 1.481; df = 2; *p* = 0.224; RMSEA = 0.035; 90% CI = [0–0.146]; CFI = 0.999; TLI = 0.993). The only variable significantly associated with expectations in the multivariate model was self-efficacy.

**Conclusions:**

The expectations scale used in our study demonstrated unidimensionality and has strong potential for clinical application. Poor self-efficacy is a potential target for intervention given its independent association with expectation. Addressing expectations directly and indirectly through self-efficacy assessment may assist in better aligning patient’s expectations with likely outcome.

**Trial registration:**

ClinicalTrials.gov NCT01620983.

## Background

Patients’ expectations of post-surgical recovery, assessed prior to knee replacement surgery have been extensively studied [1]. Because preoperative expectations have been associated with patient satisfaction following surgery [2, 3], there is strong interest in assessing patients’ expectations and potentially modifying unrealistic expectations to improve patient satisfaction [4]. However, the measurement of expectations can be complex as patients may have vastly different ideas of what they expect to be able to do and how they expect to feel after surgical recovery [1]. Additionally, other studies have shown no relationship between pre-operative expectations and satisfaction with surgery [5, 6] which have led some to speculate that variation in the association between preoperative expectation and satisfaction is complicated by the lack of psychometrically sound expectation instruments [1, 7].

As a construct, expectation may be conceptualized as a latent variable. As such, it can only be indirectly measured via the use of indicator variables. For example, the extent of pain relief and functional improvement a patient might expect following surgery are commonly used indicator variables in expectation scales developed for knee replacement surgery [2, 4, 8–14]. As of October, 2012 an estimated 25 expectation instruments for knee replacement had been described and most of these had no evidence of psychometric development or validation [1].

The most extensively developed and commonly referenced preoperative expectation scale for knee arthroplasty (KA) recovery is the Hospital for Special Surgery Knee Replacement Expectations Survey (HHSKR) [4, 11]. The original HHSKR contained 17 questions for patients to rate the importance (from “very important” to “I do not expect this”) of each scale item (e.g., straighten knee, being employed, improved psychological well-being) [11]. A later version changed the responses from an emphasis on importance to an emphasis on expectation of extent of recovery (from “complete improvement” to “not applicable”) for each scale item [4]. Scores for each item are summed to create a total score. The investigators reported on the development and reliability of the original scale [11] but did not determine if the expectation scale reflected only one dimension or multiple dimensions. None of the instruments we found examined dimensionality of the construct of patient expectation of recovery and whether it reflected a single or multiple dimensions. One could imagine that the HHSKR, for example, may actually reflect multiple dimensions related to expectation, the main construct of interest. For example, items dealing with stairclimbing, kneeling, and squatting may reflect one dimension while other items like employment and interacting with others may reflect another dimension. If multiple dimensions were found for the HHSKR, this would have important implications for scale score interpretation and would indicate the need for a sub-score for each dimension to fully interpret the scores. Because of the length of the HHSKR (17 items) which impacts clinical feasibility and because of the lack of evidence regarding dimensionality, we elected to use a shorter and more focused scale in the current study.

We recently recruited a multi-site sample of 384 persons with moderate to high levels of pain catastrophizing, an indicator of poor pain coping [15], for a randomized clinical trial of a pain coping skills intervention [16]. As part of preoperative data collection, patients completed an expectations scale [10]. Much like all other expectation scales that we found [2, 4, 8–14], the scale we used [10] had not been studied to test the measurement structure of the instrument.

One purpose of our study was to determine the extent to which the patient expectations scale reflects a unidimensional construct. We hypothesized that the scale would reflect a single construct. Our second purpose was to identify pre-operative variables associated with expectations of postsurgical recovery. We hypothesized that previously identified evidence-based predictors of preoperative expectations scale score [10, 17] (i.e., higher (worse) pre-operative Western Ontario and McMaster Universities Arthritis Index (WOMAC) Pain scores, greater comorbidity, not having assistance at home and African American race) would be associated with higher (worse) expectations in our sample. We further hypothesized that scores for previously unexplored variables of pain catastrophizing, depressive and anxiety symptoms and number of painful body regions would positively associate with KA recovery expectations (i.e., higher scores would associate with higher (worse) expectations) while self-efficacy scores would be negatively associated with expectations (i.e. higher self-efficacy scores would associate with lower (better) expectation scores). The association between pain catastrophizing and preoperative expectations was examined in one study though the expectation measure reflected a 1-month postsurgical outcome (versus outcome expectation following surgical recovery as was measured in the current study) and pain catastrophizing associated only with 2 of 4 items in the expectation scale [18].

## Methods

### Study design and setting

The study was a National Institutes of Health/National Institute of Arthritis and Musculoskeletal and Skin Diseases funded randomized clinical trial (UM1AR062800) conducted at 5 sites (Durham, North Carolina, New York, New York, Richmond, Virginia, Winston-Salem, North Carolina, and Springfield, Illinois). Patients were recruited and signed an IRB approved consent form between January, 2013 and June, 2016. The current study was cross-sectional. Only the pre-operative baseline data were used to test the study hypotheses.

### Participants

Patients were considered eligible for consenting to the study if they: (1) were aged 45 years or older; (2) had a diagnosis of knee osteoarthritis; (3) had KA surgery scheduled within 8 weeks following consent; (4) scored a 16 or greater on the Pain Catastrophizing Scale a score that was found to associate with poor WOMAC Pain outcome [19], and (5) were able to read and speak English. Patients were excluded if they: (1) were scheduled for knee revision surgery; (2) underwent another arthroplasty surgery within 6 months of the surgery of interest; (3) had a diagnosis of inflammatory arthritis (e.g. rheumatoid arthritis, psoriatic arthritis); (4) were scheduled for bilateral KA; (5) planned to undergo hip or knee arthroplasty within 6 months after current knee arthroplasty; (6) scored a 20 or greater on the depression screener [20]; and (7) scored a 2 or less on the cognitive screener [21]. A total of 4043 subjects were screened for admission to the study and 402 were enrolled. A total of *n* = 3641 were excluded, with *n* = 1694 not meeting inclusion criteria, *n* = 748 declined participation and *n* = 1199 excluded for a variety of other reasons. A total of 18 subjects who consented to participate had their surgeries cancelled for medical reasons after consent, leaving 384 subjects who underwent KA surgery subsequent to study admission.

### Data collection procedures

Patients were approached by a site coordinator within eight weeks prior to surgery and were informed about the purpose of the clinical trial. If patients expressed a willingness to participate, they read and signed an Institutional Review Board (IRB) approved consent form and then completed all required baseline data collection via an in-person interview. All patients underwent either total knee arthroplasty (*n* = 367) or partial knee arthroplasty (*n* = 17). A total of 32 surgeons performed KA surgeries across the 5 sites and the total number of patients seen by each surgeon ranged from 1 to 54.

### Outcome measure of interest

The self-reported outcome of interest was the four-item patient expectation measure [10]. This is one of the first expectation scales reported in the literature. Little information was provided on the development of the scale though the foci of the 4 items was on pain and functional improvement expectations, overall surgical success and likely complication risk. Expectations of pain, functional status and complications following KA have been incorporated in most expectation scales developed following the publication by Mohammed and colleagues [1]. The 1st item (i.e., “How painful do you expect your knee to be in one year?”) and the 2nd item (i.e., “How limited do you expect to be in your usual activities in one year?”) were each scored on a 4 point scale from not at all painful (or not limited) to very painful (or very limited). The 3rd item (“How likely will your surgery be a complete success?”) and the 4th item (“How likely will you have a knee joint complication?”) are each scored on a 0 to 10 scale. For the 3rd item, 0 equates to not at all likely while 10 indicates the patient is very likely to have a completely successful outcome. For the 4th item, 0 indicates the patient is not at all likely while a score of 10 indicates the person is very likely to have a complication. In summary, items #1, 2 and 4 are scored such that higher scores equate to worse outcomes while for item #3, higher scores indicate a better outcome. For the primary analyses, we kept the scoring as originally described. Developers of the scale did not provide a rationale for the different scaling for the four items. To provide a straightforward method for describing raw scores for the patient expectation scale, we transformed scores for item #3 so that higher scores equated to worse outcomes, much like the other items. In addition, we converted the four-point scale scores for items #1 and 2 to an 11-point score by multiplying each score by 11/4 so that each item of the scale ranged from 0 to 10. These transformed scores are summarized in Table 1. Validity has been demonstrated to the extent that higher expectations was associated with better pain and function outcome following arthroplasty [10].

**Table 1:** Characteristics of the Sample (*n* = 384)

Variable	Mean (sd) or %
Demographic	
Age	63.18 (8.03)
Sex (female)	66.9%
Race (African American)	34.90%
Education	
Less than high school	5.7%
High school graduate	22.4%
Some college	26.3%
College degree or higher	45.6%
Assistance at home (no)	12.0%
Overall Health	11.2%
Modified Charlson comorbidity score	8.64 (4.07)
Opioid use (yes)	31.3%
Number of bodily pain sites	5.58 (4.03)
Self-reported Knee Symptoms	
WOMAC Pain	11.39 (3.36)
Psychological Health	
Patient Health Questionnaire (PHQ-8)	5.87 (4.89)
Generalized Anxiety Scale (GAD-7)	5.37 (4.94)
Self-efficacy Scale	49.32 (17.74)
Pain Catastrophizing Scale	29.95 (9.27)
Expectation Scale^a^ (higher scores indicate worse expectation)	
1-year pain expectation	1.36 (1.96)
1-year activity expectation	1.57 (2.09)
1-year complete success expectation	0.57 (1.31)
1-year complication expectation	1.55 (2.47)
Total score (0 to 40)	5.05 (5.31)

^a^The four items for the expectation scale were transformed such that each item was scored on a 0 to 10 scale with higher scores equating to worse outcome expectations

### Predictors in construct validity analysis

We studied several potential correlates of preoperative expectations of postsurgical recovery. The self-reported Western Ontario and McMaster Universities Arthritis Index Arthritis (WOMAC) Pain scores ranged from 0 to 20 with higher scores indicating greater pain with activity. Because greater preoperative pain is associated with worse outcome [22], we suspected that we would find an association between pain scores and expectation scores. Reliability and validity of the WOMAC has been repeatedly demonstrated for persons with KA [23–25]. The Pain Catastrophizing Scale (PCS) and the Arthritis Self-Efficacy Scale (ASES) also were hypothesized to be associated with expectation. Pain catastrophizing quantifies the extent to which a person amplifies their pain symptoms, feels helpless when experiencing pain and ruminates about their pain. The PCS is a 13 item scale ranging from 0 (no pain catastrophizing to 52 (most severe pain catastrophizing) [15]. A substantial literature supports both the psychometric and prognostic importance of the PCS for patients undergoing KA [15, 19, 26, 27]. Pain catastrophizing has been shown to be associated with The 8-item ASES is a validated measure of a patient’s beliefs in the ability to control pain and functional difficulty associated with arthritis [28]. Scores for the ASES range from 8 (lowest self- efficacy to 80 (highest self-efficacy). Persons with higher self-efficacy were expected to have higher expectations of outcome.

Race was dichotomized to African American subjects or all other subjects. African Americans are less likely to undergo KA and are less trusting of healthcare recommendations regarding KA surgery [29]. We used the validated modified Charlson comorbidity index to quantify extent of comorbidity [30]. We suspected that patients with a greater morbidity burden would have lower expectations than persons with less comorbidity. The previously validated Patient Health Questionnaire (PHQ-8) [20] and the Generalized Anxiety Scale (GAD-7) [31, 32] were used to quantify extent of depressive and anxiety symptoms, respectively. The PHQ-8 is scored from 0 to 24 and the GAD-7 is scored from 0 to 21 with higher scores indicating more severe depressive or anxiety symptoms. Because both anxiety and depressive symptoms are negatively associated with a person’s level of optimism [33], we suspected that higher levels of either depressive symptoms or anxiety would negatively associate with expectation. Number of bodily pain sites (e.g., low back, neck, right shoulder), ranging from 1 to 16 sites were determined [34]. Because we were interested in musculoskeletal widespread pain and not fibromyalgia per se, we did not include the jaw and abdomen items from the original scale. We suspected that persons with greater numbers of bodily pain sites would have lower expectations because the KA surgery would very likely have no effect on pain from these other sites. Finally, based on prior evidence [17] we suspected that not having assistance at home would associate with worse expectations.

### Covariates

To account for other variables that may be associated with both expectation and our predictors or interest, we also adjusted for patient age, sex, opioid use (yes or no), and education (less than high school, high school graduate, some college, college degree).

### Data analysis

Confirmatory factor analysis was used to test the measurement structure and baseline variables associated with the expectancy scale. We tested two measurement models: a one-factor model with and without the method factor and compared them during the model selection process. The best fitting model was subsequently used in the validation of the instrument using structural equation modeling. The measurement model specification with and without the method factor was motivated by the difference in item stems (“how likely” versus “how painful/limited”) as well as the response format difference between two sets of items (0–3 versus 0–10) that might potentially yield larger covariances between items with the same item characteristics. We used the M-1 method factor specification with unit factor loadings as suggested by Eid [35]. Trait and method factors were orthogonal in the measurement model with the method effect.

To account for clustered sampling design (patients nested within surgeons), we used the random intercept model to account for correlated data. Items with a 0 to 3 scoring format (later transformed to a 0 to 10 scale) were fitted as ordinal categories in all models because categorical outcome variables (factor indicators) violate the multivariate normality assumption.

Using the appropriate measurement model selected using confirmatory factor analysis, a measurement invariance test was conducted between the gender groups followed by a structural model to identify predictors of the latent expectation construct. Potential predictors included demographic characteristics, preoperative pain, assistance at home, pain catastrophizing, depressive symptoms, anxiety, and number of pain sites, self-efficacy, and opioid use. Each predictor was testing using an alpha level of 0.05. Mplus software (version 7.4) was used in model fitting.

## Results

We consented a total of 384 patients who underwent KA surgery. The average age of the sample was 63.18 (standard deviation/sd = 8.03) years, 34.9% were African American and 66.9% were female. The average pain catastrophizing score was 29.95 (sd = 9.27) and is indicative of persons with moderate to high levels of pain catastrophizing [15]. The characteristics of the sample are reported in Table 1.

### Dimensionality of the expectation scale

Descriptive statistics along with skewness and kurtosis estimates for the four items of the expectations scale appear in Table 2. The unidimensional latent variable model without a method factor was inconsistent with the data (χ^2^ = 24.962; df = 2; *p* < 0.001; RMSEA = 0.173; 90% CI = [0.116–0.236]; CFI = 0.942; TLI = 0.826). A method factor had a superior model fit (χ^2^ = 1.481; df = 2; *p* = 0.224; RMSEA = 0.035; 90% CI = [0–0.146]; CFI = 0.999; TLI = 0.993). The statistical difference between the two models was significant (Δχ2 = 28.11; df = 1; *p* < .001) indicating that the unidimensional expectation scale with a method factor was the best fitting model. Incorporating the method factor assures that the unidimensional Expectancy factor is not confounded with this source of systematic error (bias).

**Table 2:** Score distributions, skewness, kurtosis and of the four items in the expectations scale

Variable	Minimum (% of sample)	Maximum (% of sample)	Mean (sd)	Skewness (SE)	Kurtosis (SE)
Pain	0 (64.3)	2 (5.2)	0.59	1.13^***^ (.13)	.27 (.25)
Limitations	0 (60.2)	2 (7.3)	0.63	.99^***^ (.13)	−.08 (.25)
Success^a^	0 (72.7)	10 (0.5)	1.32	−3.70^***^ (.13)	17.74^***^ (.25)
Complications	0 (56.3)	10 (2.3)	2.47	1.85^***^ (.13)	2.84^***^ (.25)

^a^This item has been reverse coded such that that higher scores equated to worse outcomes, much like the other items in the scale

^***^ *P* < .001

**Fig. 1:**
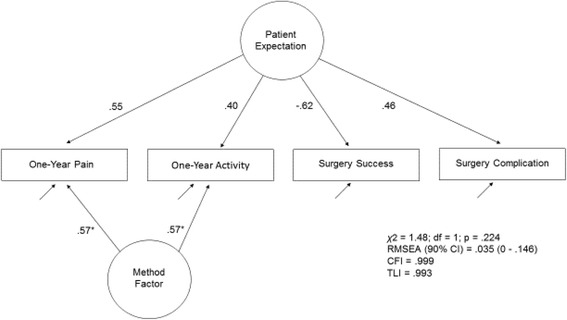
The latent construct, Patient Expectation, is connected via arrows to four indicator variables that comprise the latent construct. These indicator variables are the individual items of the expectation measure (i.e., One-year pain, One-year function, Surgical success and Surgical Complication expectations). Each arrow also is labeled with a coefficient that describes the strength of association between the latent construct of Patient Expectation and the observed variable. The One-year pain and One-year function variables are connected via arrows from a Method factor

The graphical representation of the measurement model with standardized factor loadings appear in Fig. 1. Method factor loadings are set equal for the purpose of model identification. The coefficients, as expected, reflected positive associations between the latent construct of Patient Expectation and the indicator variables of one-year pain, one-year limitation and surgery complication expectations. The Surgery Success indicator variable, as expected, was negatively associated with the latent construct because of the reverse coding of this item in the original scale.

### Construct validity evidence

Measurement invariance for sex based differences was assessed. The model with scalar invariance restriction (equality of factor loadings and item thresholds between the groups) fit the model as well as the configural invariance model (factor loadings are freely estimated in both groups): χ2 = 7.29; df = 7; *p* = .399; RMSEA (90% CI): .015 (0–.091); CFI = .999; TLI = .998. Based on the rule of parsimony, the results support the measurement invariance between males and females.

**Fig. 2:**
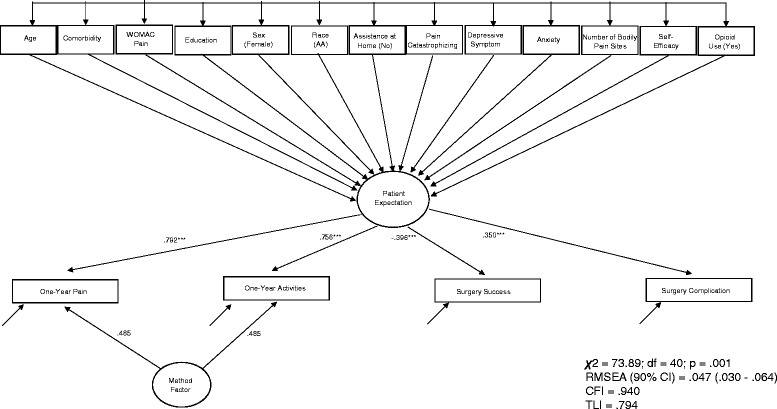
Standardized coefficients are shown. Estimated coefficients describing the relationships among predictors of the latent patient expectation variable are presented in Table 3. Path coefficients from the predictors and the latent patient expectation variable are given in parenthesis when the model included only one predictor

Structural equation modeling was used to predict the latent expectation variable using all predictor and covariate variables (see Fig. 2). Standardized and unstandardized regression coefficients for each variable in both the univariate and multivariate models appear in Table 3. For the univariate analyses, comorbidity, WOMAC Pain, depressive and anxiety symptoms, number of bodily pain sites were positively associated (*p* < 0.01) with expectations such that higher (worse) scores in each of these predictors were associated with higher (worse) expectations. Opioid use was the only covariate associated with expectations (*p* = 0.043). Self-efficacy was negatively associated (*p* < 0.001) with expectations such that higher self-efficacy scores were associated with lower (better) expectations. Only one variable, self-efficacy, was associated with expectation after adjustment for all other variables in the model. Persons who scored higher on self-efficacy tended to score lower (better) on the Patient Expectation latent construct, as hypothesized. No other variable was associated with the latent construct of expectation after adjusting for all other variables in the model. A correlation matrix of all 13 predictors of the expectation latent variable appears in Table 4.

**Table 3:** Structural Equation Modeling results for associations between preoperative expectation score and covariates

	Simultaneous Estimation			One Predictor at a Time		
Predictor Variables	Unstandardized Beta (95% CI)	Standardized Beta	*P* value	Unstandardized Beta (95% CI)	Standardized Beta	*P* value
Age	−.001 (−.011–.009)	−.007	.890	−.006 (−.016–.004)	−.082	.250
Comorbidity	.022 (0–.044)	.111	.051	.042 (.020–.064)	.247	<.001
WOMAC Pain	.011 (−.021–.043)	.045	.514	.034 (.012–.056)	.185	.002
Education	−.021 (−.127–.085)	−.025	.699	−.035 (−.111–.041)	−.058	.344
Sex (female)	−.198 (−.418–.022)	−.118	.072	−.096 (−.248–.056)	−.082	.177
Race (African American)	.118 (−.126–.362)	.071	.333	.119 (−.115–.353)	.099	.309
Assistance at home (no)	−.100 (−.426–.226)	−.040	.537	−.158–.412–.096)	−.092	.198
Pain Catastrophizing Scale	.004 (−.012–.020)	.043	.634	.011 (−.001–.023)	.169	.053
Depressive symptom Scale	.015 (−.013–.043)	.090	.294	.036 (.020–.052)	.305	<.001
Anxiety Scale	.012 (−.010–.034)	.073	.300	.036 (.020–.052)	.299	<.001
Number of bodily pain sites	.008 (−.026–.042)	.043	.608	.030 (.006–.054)	.209	.008
Self-Efficacy	−.007 (−.013 - -.001)	−.146	.020	−.008 (−.012 - -.004)	−.262	<.001
Opioid Use (yes)	.094 (−.082–.270)	.055	.284	.154 (.004–.304)	.115	.043

**Table 4:** Relationship between the predictors of the latent Patient Expectation variable

	1	2	3	4	5	6	7	8	9	10	11	12	13
1. Age	1												
2. Comorbidity	−.006	1											
3. Womac Pain	−.176**	.283***	1										
4. Education	.137*	−.136**	−.229***	1									
5. Sex (Female)	.034	.055	.102*	−.024	1								
6. Race (AA)	−.223***	.165**	.219**	−.281***	.039	1							
7. Assistance at Home (No)	.023	−.003	.014	−.008	−.021	.052	1						
8. Pain Catastrophizing	−.191**	.182***	.427***	−.269***	−.017	.208***	.017	1					
9. Depressive Symptom	−.197***	.350***	.291***	−.145**	.093*	.064	−.047	.260***	1				
10. Anxiety	−.224***	.308***	.258***	−.141**	.121***	.080	−.079	.309***	.703***	1			
11. Number of Bodily Pain Sites	−.059	.407***	.339***	−.153***	.146*	.183***	.014	.159***	.331***	.336***	1		
12. Self-Efficacy	.228***	−.234***	−.252***	−.032	.002	−.042	.087	−.206***	−.368***	−.337***	−.215***	1	
13. Opioid Use (Yes)	−.198***	.295***	.206***	−.167***	.032	.237***	−.010	.084	.183***	.176***	.210***	−.035	1

Note. Entries are correlation coefficients estimated from the model presented in Fig. 2. *p < .05; **p < .01; ***p < .001

## Discussion

Expectations of postsurgical recovery, assessed preoperatively, are considered by orthopaedic surgeons to be an important and commonly used prognostic indicator of patient outcome and patient satisfaction after KA [1, 3, 6]. If a patient’s expectations are too high (e.g. if a patient expected to participate in recreational running for moderate to long distances after surgery) surgeons would typically intervene to enhance chances of long-term implant survival. If the patient’s expectations are too low (e.g., if a patient expected at least moderate pain after the surgery) the surgeon could provide information on the most likely pain level following surgery. Despite a clear rationale and need for assessments of patients’ preoperative expectations, psychometrically sound instruments for measuring patient expectations are lacking [1, 7].

We evaluated the measurement structure of the expectation scale [10] collected from a large prospective sample of patients with moderate to high levels of pain catastrophizing, a characteristic associated with poor outcome [19, 26, 36]. We found that the scale reflected a unidimensional construct suggesting that clinicians can be confident that a single score reflects the entire scale. To our knowledge, our study is the first to provide confirmatory factor analysis evidence of unidimensionality for a KA expectations scale.

Our findings related to correlates of expectations were unexpected. Only self-efficacy associated with expectation scale scores in our multivariate model. Several other baseline variables shown to associate with expectations in other studies (i.e., pre-operative WOMAC Pain scores, comorbidity) were associated with expectations in our study but not when adjusted for other variables in the model. Additionally, we hypothesized that pain catastrophizing, depressive symptoms and number of painful body regions would be positively associated with expectations in our sample, and all were associated but only in univariate analyses. We suspect the truncated range of scores for the PCS may have limited our ability to detect an association between pain catastrophizing and expectations. We specifically recruited persons with moderate to high catastrophizing and excluded persons with either a score of 0 or only mild catastrophizing.

Prior work [10, 17] only identified univariate associations between predictors and latent variable expectations score for some of the variables we studied. Ours was only the second study, [17] that we found to generate a multivariate model of correlates of expectations. Hepinstall and colleagues [17] examined 1943 patients preparing for KA and found that age, assistance at home, history of prior joint arthroplasty, SF-36 General Health, KOOS Quality-of-Life scores associated with expectations in a multivariate model. We did not find that either age or assistance at home was associated with expectation.

Hypothesized associations between expectations and potential correlates were obtained in prior studies using a variety of expectations measures on a broad spectrum of patients undergoing KA [3, 6, 10, 17]. Our study was conducted on a homogenous group of patients with moderate to high levels of pain catastrophizing and it is possible that this phenotype of patients explained our findings relative to these other studies. We found few studies that explored associations between expectations and a variety of psychological distress measures [3]. While we expected that persons with higher levels of pain catastrophizing and depressive symptoms would likely have lower expectations because of their distress, only univariate analyses support this assumption. Similarly, we thought it reasonable to hypothesize that persons with pain in more body regions would have lower expectations because the surgery only targeted a knee and not these other body regions. This hypothesis also was only supported in our univariate analysis.

The only hypothesized association we found in our multivariate model was between expectation and patient self-efficacy. The higher a patient’s self-efficacy, the better the preoperative expectation. Our data suggests that self-efficacy is an independent correlate and is moderately associated with expectations. Self-efficacy, the belief that one can control both pain and functional challenges associated with arthritis and KA can affect expectations negatively if the patient’s self-efficacy is poor. Addressing reasons for poor self-efficacy may assist in better aligning expectations with expected outcome leading to better results and potentially improving patient satisfaction, given substantial evidence indicating that about 20% of patients are dissatisfied with their outcome and a similar proportion have only slight or mild pain following recovery [37, 38]. Alternatively, in patients with very high self-efficacy, expectations may be unrealistically high and the surgeon may need to temper a patient’s expectations. Only about 40% of patients are pain-free following KA recovery and over 60% of our patients expected to be pain free after recovery. Self-efficacy was the variable in our study that most strongly associated with expectation in both univariate and multivariate analyses and therefore is the likely variable to inform interventions designed to align expectations with most likely outcome.

Our study had some important limitations. Our study design was cross sectional and the patients we studied had moderate to severe pain catastrophizing. This population may respond differently to expectations scales as compared to the general total knee arthroplasty population. Interestingly, the expectations scores in our sample suggested very high expectations, with mean scores of 5.05 (sd = 5.31) on a 0 to 40 scale with lower scores indicating higher expectations. Given the elevated risk of poor outcome for persons with moderate to high catastrophizing [19, 26, 27, 36], it is likely that many in our sample had artificially inflated expectations. Approximately 63% of our sample expected complete pain relief, 60% expected complete return to daily activities, 72% expected complete success following the procedure and 56% expected no complications. A substantial ceiling effect raises concerns [39] regarding the interpretation of scores and likely limited our ability to identify correlates of expectation. However, scores of 0 on the expectations scale are potentially informative because they indicate the patient’s expectations may be too high. Patients’ pain and function are substantially improved, on average, following KA surgery but some residual discomfort and functional compromise is very common [22, 40, 41]. Finally, we did not compare our expectation scale to another scale designed to measure expectations and this would have provided another approach to assess construct validity.

## Conclusion

We found that the patient expectation scale [10] is unidimensional. Users can be confident that a single score reflects the entire scale. Additionally, we found that of the several baseline variables hypothesized to be associated with expectations, only self-efficacy was found to be significant in our multivariate model. Patients with poor self-efficacy scores may benefit from interventions to both improve self-efficacy and better align expectations with likely outcome. The expectations scale score was substantially influenced by ceiling effects but these effects do not necessarily preclude potentially worthwhile application of the instrument. Scores of 0 on the scale inform the surgeon that expectations may be unrealistically high, particularly in patients with moderate to high pain catastrophizing like those in our sample.

## Abbreviations

ASES: Arthritis Self-Efficacy Scale
GAD-7: Generalized Anxiety Scale
HHSKR: Hospital for Special Surgery Knee Replacement Expectations Survey
IRB: Institutional Review Board
KA: Knee arthroplasty
PCS: Pain Catastrophizing Scale
PHQ-8: Patient Health Questionnaire
WOMAC: Western Ontario and McMaster Universities Arthritis Index
